# The Role of the Digital Economy in Rebuilding and Maintaining Social Governance Mechanisms

**DOI:** 10.3389/fpubh.2021.819727

**Published:** 2022-01-14

**Authors:** Fengjuan Niu

**Affiliations:** Department of Public Security, Henan Police College, Zhengzhou, China

**Keywords:** digital economy, social reforms, sustainable digital economy, social governance, governance mechanism

## Abstract

The digital transformation has impacted society at different levels, mainly on the economic and governance levels. This paper investigates the impact of the digital economy on social governance mechanisms. Additionally, it captures the indirect effects or mediating forces such as social reforms and a sustainable digital economy. The study followed a positivism philosophy, and it is survey research influencing cross-sectional study. The unit of analysis in the current paper was employees from four different professions as economists, financial analysts, managers, and teachers. The random sampling technique was used as a sampling type, and a questionnaire was used for data collection. Structural equation modeling (SEM) was carried out as a data analysis technique. The research findings revealed that the digital economy has a favorable impact on the social governance mechanism. Likewise, the digital economy positively affects social reforms and a sustainable digital economy. Social reforms also proved to link with a sustainable digital economy positively. The output of the indirect effects and structural model confirmed that social reform played a partial mediation role between the digital economy and sustainable digital economy. Moreover, a sustainable digital economy confirmed a partial mediation between the digital economy and the social governance mechanism. Finally, analysis confirmed a serial mediation among digital economy, social reforms, sustainable digital economy, and social governance mechanism. Therefore, policymakers and government agents should improve the digital economy to have a strong social governance mechanism.

## Introduction

The numerous adoptions of digital technologies in recent times have stimulated the huge transformational process called digitization. Digitization has helped society to process, produce, transfer, and share all sorts of data and information. However, digital transformation has not happened overnight or is a one-time event despite its fast pace. According to Avotra et al., it has occurred gradually and is driven by three different waves of technological revolution and innovation (1). The first wave of digitization is supported by the adoption and introduction of refined technologies such as information management systems, automatic reporting and monitoring systems for business performance, broadband, voice telecommunications, including fixed and mobile. All these technologies have helped to access information remotely. Furthermore, the advent of the internet and its conforming extensions such as e-markets and search engines initiated the second wave of digitization. The second wave enabled the big web of dot coms to connect the consumers and enterprises at a new level for purchasing, selling, and distributing services. Consequently, the second wave transformed into the third one entailing the more advanced technologies such as the internet of things (IOT), artificial intelligence, robotics, and sensors. All this advanced development focused on enhancing the information processing, automating the daily mundane tasks within the business organizations as well governments, and increasing the accuracy of the decision-making process for organizations (2–4).

Berger and Frey (5) suggest that digital transformation has impacted society at different levels, mainly on the economic levels. The automation of different business operations such as increasing production, reducing costs, and enhancing the operational frameworks have added a huge sustainability benefit to the businesses. The digital economy has offered novel opportunities for businesses and the job market. The extensive and diverse range of services the digital economy offers has created numerous new jobs that have impacted both entrepreneurial and employment markets. The digital economy uses a huge amount of data and information for its operational framework that has helped to deliver the same public services such as health and education more efficiently. The sustainable digital economy has also impacted the social governance mechanisms by enhancing the quality of interactions between governments and their citizens (5).

Lastly, the other major and big impact of the digital economy is social relationships and human behaviors by providing social inclusion and shared communication opportunities. However, Goos et al. has highlighted the other side of this coin that reveals that the digitization of businesses does have some disadvantages (6). For example, the internet scams and cybercrimes, labor interruption, evaporation of companies, and social isolation at individual levels. These disadvantages challenge the regulators and policymakers to understand the ever-changing process of digitizing the economy more comprehensively. The digital economy is in constant transformation and innovation diffusion. By understanding the patterns of transformations, the information communication and technology regulators and policymakers can predict the changes resulting from gradual waves of digitization and technological progress, leading to a better assessment of the digital economy. Hence, to achieve a sustainable digital economy, digitization should be perceived as two processes at once: research and development and evolution through innovation among the consumers, enterprises, and governments adopting technology.

Moreover, it is important to distinguish between both processes because the former one (technological progress) is way ahead of the latter one (diffusion). This means there could be a significant gap between product accessibility and impact. For example, in the 1940s and 1950s introducing computing into businesses did not yield a noticeable impact on productivity. It took almost 40 years (1990) for the significant impact to emerge.

Subsequently, the internet has boomed in the mid of 1990s resulting in an expanded digital space that has changed how businesses generally operate along with the advanced methods of transactions between consumers and businesses. This is when computers have emerged globally and abundantly involved in the world economies. The new world economy has become reliant on internet technologies like never before. It has been explains that in 2015 more than 75% of Americans were reported as regular users of the internet as compared to in 2000 with only 44% users (3). According to the U.S Department of commerce reports, there are is still a little doubt about to what extent the digital platforms impact American businesses and their part in enhancing the overall economic growth of the country. Hence, measuring the impact of the digital economy is essential for evaluating the overall economic growth since the continuously increasing reliance of consumers and businesses on digital platforms and services (7, 8).

The digital economy has been sustainable at several levels. Sturgeon (9) thinks that the main characteristic of the digital economy is that it frees businesses from relying on geographical locations resulting in removing the better location from the list of competitive advantages. This however shifts the business's complete reliance only on digital technology such as mobile devices, websites, smart contracts, and cloud computing (9). Bag et al. observe that the digitalization of the economy is a central factor for achieving a sustainable and competitive economy overall (10). Yun et al. (11) describes this transformation as the destruction of traditional business models and industries resulting in blurred boundaries between technology and business, technology has proven itself to be the catalyst for business transformation and strategy. The organizations have a new perspective of technology and are stimulated to rethink their operating models and the role of technology in the organizational structures (11).

The impact of the digital economy has been studied many times before and is not a new idea. For more than two decades, the department of commerce of different countries, the Bureau of Economic Analysis (BEA), and other agencies have been researching and publishing multiple reports measuring the impact of the digital economy on different social and political governance mechanisms. The reports on the digital economy can be dated as far as 1998. The US census bureau in 2001 published a report using the same rationale that is currently used to measure the impact of the digital economy. Moreover, the US Department of Commerce in 2016 formulated a board of advisors to run a Digital Economy Board (DEB) consisting of leaders from both academia and industry. These members offer a diverse range of professional knowledge and experience on the digital economy and its relation with economic policies. In their first report, DEBA has recommended different ways to measure or monitor the impact of indicators of digital economy such as productivity and GDP, including the extent of digitalization of different economic sectors (12).

It is to be noted that the significant characteristic of the digital economy is not technology anymore but innovation. The internet provides an opportunity for innovative minds to develop new ways to solve old problems, unlike oil and chemical sectors that have old and outdated environmental and social concerns when pressured by stakeholders. However, on the other side, the e-business models include these concerns on the very primary levels. The digital economy understands the urgency of the issues rather than delaying them to the point when it becomes a burden or challenge for the current way of doing things. Hence, any fast-changing and young sector can adapt these digital measures more easily than any old organization trapped in traditional mindsets. The digital economy with the right vision, intelligent policy, and creative imagination can help to achieve sustainability (13).

The digital economy has given birth to sustainable E-commerce that has changed the power balance between consumers and businesses. The most major benefit for consumers is the availability of cheap goods and services as the prices can be compared with a single click. However, consumers aren't the only stakeholders in the whole framework that are befitted from e-commerce. There is no doubt that the internet provides inclusive and responsible business models, but it also requires establishing clear ethical criteria. The current digital economy is often under suspicion by consumers due to unsolved privacy and security issues related to online transactions. As the digital economy advances as a more established and sophisticated model the interactions including both business to consumers or business to business face a diverse range of issues including both social and environmental impact of the products and services being sold online (13).

Since the internet already blurs the traditional boundaries in almost all sectors including breaking the social class difference in terms of information accessibility and bringing the government out of its walls, the current paper aims to explore the more complex phenomenon of the internet known as the digital economy and its impact on the sustainability, social reforms as well as social governance mechanisms. The current research is well-aware of the fact that the web is a major source of building new partnerships and alliances. Hence, to build a sustainable digital economy, the world needs to connect the dots. All the major Dotcoms, including Dot organizations and Dot governments, require to come on the same page to share ideas and innovate new solutions for existing problems to achieve sustainability in every economic sector. It is important to measure the relationship among digital economy, sustainability, social reforms, and governance mechanisms as the digital economy evolves, it appears to be more difficult for businesses to maintain their market leadership. The research and development in this area are important because it doesn't matter how well a business can keep the pace of innovation in different sectors of the economy it can still face serious problems if stayed under-researched. In short, the digital economy is changing so fast that it seems impossible for businesses to keep a sustainable advantage. The only way for any company to keep its competitive advantage is to keep up with the digital transformation of the economy through research development.

## Review of Literature

### Digital Transformation and Sustainable Digital Economy

The digital economy refers to utilizing digital tools and advanced technologies such as mobile applications, social networks, and e-commerce into normal businesses (9). It consists of the transformation of an organization's business strategy toward adapting the innovative technologies and applying digitalization to increase its value production. This strategic transformation helps to support innovation by providing detailed market insight and consideration toward the newly conceived ideas (14). The previous studies suggest that for increasing a business's production value, the organizational strategies equally matter as much as the adaption of new technologies. Hence, a profound analysis is required to explore the possibility of redesigning existing business models to keep up with the digital transformation to achieve a sustainable digital economy. This can lead to creating organizations with better performances and competitive advantages. Which can impact the whole economy positively and add to the benefits for both consumers and businesses as some past studies have already revealed (15). Kane et al. (16) have explained the impact of organizations adoption of new technologies as a contribution to the social reforms and well-being of the respective society. A digital economy enables businesses to survive in an era of ever-changing consumers and supply demands by adapting to the latest digital information tools (17). Any digital economy can lead to sustainability when its firms have the potential to have an in-depth up to date understanding of digital innovation which is derived from the internal research and development sources of the economy (16, 18, 19). Therefore, to establish a sustainable digital economy, every organization must consider taking its business to the available digital platforms enabling optimization, innovation, consumer interaction that can eventually lead to a better work environment and transformed business context (15). The sustainable digital economy is not solely derived from the link between digital platforms and technologies but also depends on the organization's speed of innovation and adaption. Thus, before anything, the digital adaption in the overall economy is the enabler of the sustainable digital economy. Subsequently, the digital upgrades must fit every individual business model present in the respective economy to achieve a sustainable digital economy and innovation (20). Under the context of the above argument supported by existing research studies, a positive link between digitalization and a sustainable digital economy can be made. Hence, it leads to form following hypotheses:

***H***_1_*: Digital economy has a positive impact on a sustainable digital economy*.

### Sustainable Digital Economy and Adaptive Governance

Generally, every government is accountable for adapting the best suitable digitalization governance strategy according to its country, culture, economic needs, and values. However, despite the deployed governance strategy within an existing government, a sustainable digital economy will always require reconsideration of the governance mechanisms and redesigning of more flexible and adaptive government mechanisms. These redefined social governance mechanisms enable stakeholders in both government and society to actively regulate their best norms and codes of conduct to acquire the advantages of digitalization without experiencing unnecessary losses or risks. To be precise, it will become inevitable for governments, in alliance with other stakeholders from academia, industry, the general public, and NGOs to actively participate to improve and implement policies that can guide the digital economy to meditate technological benefits against the possible environmental and social disrupting factors especially as both information and experiences tend to move toward advancement with time.

Linkov et al. shows three different types of governance strategies for a sustainable digital economy presented in the Organization for Economic Co-Operation and Development (OECD) (21). These three strategies include (i) laissez-faire (industry-driven approach), (ii) precautionary strategy (pre-emptive strategy on the part of government), and (iii) stewardship (an “active surveillance” method by government agencies) to mitigate the risks of digitalization while supporting the innovation in private sectors. Despite the given governance strategies, adaptability is required to deal with risks and threats related to digital sustainability as formulated by Sustainable Development Goals (SDGs) (22). Adaptive governance is defined by Trump (2017) as the adaption of rules and practices that can regulate the incorporation of new technologies and data to balance the benefits and risks of given digital activity (23). It is possible that this balance might be achieved within the three proposed governance strategies. For instance (24), adaptive governance can be formulated through legislation to assess the risks and evaluate the present regulatory structures with the help of progressing space and loss. In this particular scenario, every regulatory hard law must offer clauses for routine amendments for risk management. Similarly, adaptive governance can also be framed willingly through voluntary measures taken by majorly affected stakeholders and can give birth to soft laws based on flexible clauses of the code of conduct that can be changed when faced with challenges posed by digitalization. Generally sustainable digital economy stimulates industry, academia, and private stakeholders to evaluate the digital services and examine the threats posed by digitalization as well as improve and propose effective governance strategies to maintain sustainability. Irrespective of the type of governance strategy in practice, every stakeholder from government and industry benefits from adaptive governance, not in terms of social and environmental sustainability but also provides a helpful and relevant governing manual. Hence, studies show that adaptive governance stimulated by the digital economy is capable enough to deal with the modern challenges faced by sustainable digital economies such as privacy breaches, transaction scams, cybersecurity threats, and stealing data (25–27).

Another governance framework that deserves consideration is Fountain theoretical model of technology enactment framework, which is based on the literature on neo-institutionalism, network organizations, governance, and bureaucracy (28, 29). It has offered some theoretical understanding of the governance framework that can work around the digitalized economies. The framework explains the interaction taking place among technology, organizations, and institutions. Fountain (28) argues that “Information technologies are not so much implemented or applied but enacted by decision-makers.” This gives rise to e-governments that can deliver services to businesses and citizens while providing law and security for the digital economy. According to Gauld and Goldfinch, the concept of e-government became part of public administration as the private sector adopted digital technologies into business and commerce (30). These e-governments with their commercial and technical features, have become “Government 2.0” in many countries (31). Despite the fact that Fountain framework has provided strong foundational support in understanding governance around the digital economy but it still has shortcomings, For example, the empirical case studies used by Fountain (28) to test her theory were mostly limited to organizations from the federal government of United States. This creates a research gap that suggests further empirical research on understanding the impact of the digital economy on governance mechanisms. Hence the current study offers the following hypotheses:

***H***_2_*: Digital Economy has a positive impact on governance mechanism*.
***H***_**3**_*: Sustainable digital economy has a positive impact on social governance mechanisms*.
***H***_**4**_*: Sustainable digital economy mediates the relationship between the digital economy and social governance mechanism*.
***H***_**5**_*: There is a serial mediation that exists between the digital economy and social governance mechanism*.

### Delivery of Research and Education by E-Government in the Digital Economy

The United Nations have set multidimensional approaches to evaluate the development of e-governments of different state members. These multidimensional approaches focus on the quality and standard of the online services, social, and educational factors including human capital, and telecommunication infrastructure. Moreover, the United Nations also focuses on evaluating e-participation from three different perspectives: sharing information, consultation, and engagement on decision-making processes by governments and citizens. This approach highlights the importance of social factors such as citizen participation in the development of e-governments established for digital economies. Research indicates that e-participation is the major key factor in attaining sustainable levels of e-democracy in a nation, leading to a sustainable digital economy (32). This indicates that the development of both sustainable digital economy and government is not dependent on a single factor but multiple factors including telecommunication infrastructure, legislation, and regulatory atmosphere, quality information, and most important social and economic reforms as well as management and engagement characteristics of citizens. The other factors that influence the adoption of the digital economy and government are demographics, skill sets, and socio-economic backgrounds of the citizens (33). Furthermore, Zhao (34) has also found culture as a major factor to influence the development of the digital economy and governments. For example, another study by Zhao et al. (35) has found that the 26 different European countries with higher power distance rates tend to have lower rates of adoption of e-governments. However, countries with high levels of individualism and continuous cultural transformation are faster to adopt a digital economy as well as government. Hence, the study has concluded that social factors such as cultures directly impact the adoption of governance mechanisms while the digital economy has a moderating effect on this direct relationship between governance and culture.

***H***_**6**_*: Social reforms mediates the relationship between the digital economy and sustainable digital economy*.

Furthermore, the other social factors such as the education sector and research and development also influence the digital economy and value transformation. For example, teachers are playing a major role in changing the nature of consumers by creating a novel type of studentship in the existing education system. The education sector of the current era is taking full advantage of the digital technologies and as well as unique networking platforms to involve all direct and indirect stakeholders in the educational process (36). Marz et al. have termed this phenomenon as “network interaction” including teachers, students, parents, and other educational stakeholders. This has enabled traditional educational frameworks to be more flexible and open toward welcoming new developments. Moreover, it has allowed the educational stakeholders to consider the psychological and physical characteristics of its consumers while designing a digital education system (37).

This updated transformation of the education system has given birth to new social reforms focused on training a better staff to lead people who require to develop in accordance with the ever-changing demands of the international labor markets in the future. Subsequently, the international educational market directly influences the quality of international labor markets and global economic development. Hence, the digital education system has become foundational for the advancement of the digital economy. The world has realized the importance of this particular educational transformation and much work has already been done in training engineers and scientists to enhance the quality of research and development for the future of the digital economy. This provides a new world view reliant on the technology, innovation, and transformation of already existing social institutions. The social reforms of modern society are either derived from artificial intelligence, machine learning and robotic or are dependent on the three. In their study, Zheng et al. (38) observe that the current post-industrial society requires a knowledge-based economy sustained through technology and smart human capital. The study highlights the huge gaps between the needs of a group and an individual. However, researchers have identified this gap for a while now due to the advent of smart cities and the Internet of Things (IOT). The current digital social structure is going to completely change the nature of human capital and social reforms related to the role of education and science in our society (38).

According to the research by Chai, dozens of Asian, American, and European countries have digital development of education and human capital as their prime focus to develop a sustainable digital economy (39). These countries are focused on developing a digital economy supported by smart human capital equipped with the use of technology and science, technology, engineering, and mathematics (STEM) education. By strengthening the education science and research development, the countries are addressing the major problems faced by economic sectors as it directly impacts the development of the digital economy, governance, and human capital. Hence, studies show [e.g., (24, 37, 38)] there are multiple influences, including the roles of policymakers, public officials, and social factors such as legal and cultural norms and the educational and political contexts that impact the sustainability of the digital economy and government. Hence the current study hypothesizes:

***H***_**7**_*: Digital economy has a positive impact on social reforms*.
***H***_**8**_*: Social reforms have a positive impact on a sustainable digital economy*.

**Figure 1:**
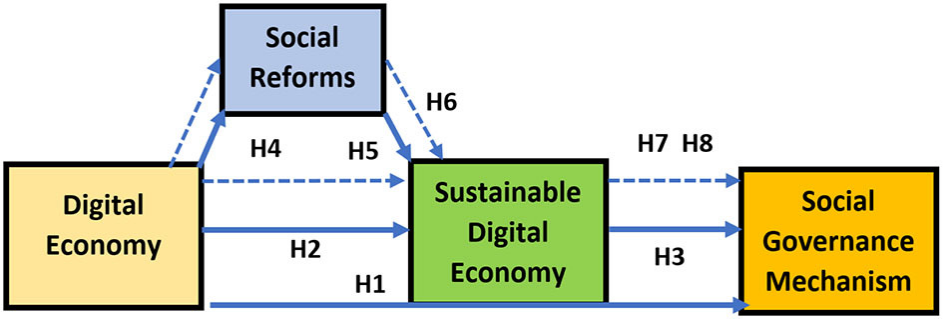
Theoretical framework.

Based on the above literature and hypothesis following framework has been formulated (see Figure 1).

### Methodology

The positive philosophy supports the current Research Topic. The study is a survey-based study with a cross-sectional aspect because the data was obtained at a specific moment in time. The survey study yields estimate that are cost-effective, dependable, and accurate. A self-administered questionnaire was used in the current research to collect data for the survey. Professionals from different professions were used as the unit of study. The paper used a random sampling technique, which means that every participant in the population has an equal chance of being chosen. As a result, the biasness of responses is minimized. The study had a total sample size of 381, however, only 450 questionnaires were disseminated across the public, with 69 being screened out during the screening procedure. The data was gathered through social media networks, and the questionnaire was created using Google Forms. Google-Forms provided an excel sheet of replies, which was then imported into Smart-PLS for structural equation modeling (SEM).

### Measurement

The paper follows already used measurement scales for each construct in the research model. There were a total of 18 items for four constructs in the model. The digital economy was measured through three items and the scale was adapted from (40). The construct of social reforms was measured through a seven-item scale and adapted from (41, 42). The scale for a sustainable digital economy was taken from (43) and it was based on four items. Finally, the social governance mechanism was taken from (44) and it was measured with four items scale. All measurement scales were modified marginally but it did not change the overall meaning where each measurement or question or item was asked on 5 points Likert scale from (5) strongly agree to strongly disagree (1).

### Data Analysis Approach

The proposed conceptual model was investigated utilizing the Smart-PLS version 3.3.3 application in this study. There are two aspects to the method: (i) measurement model evaluation and (ii) structural model evaluation. As advised by previous studies, these two processes are typically trade-offs when using the single-step technique (45, 46). The structural model evaluation emphasizes the link between model variables, whereas the measurement model evaluation demonstrates how all model variables are assessed. The consistency of indicators and constructs in the research model is included in the estimation of the measuring model. Furthermore, it encompasses both discriminant and convergent validity. To assess the constructs' and indicators' reliability and validity, certain estimations are used. Factor loadings (FD), construct reliability (CR), and Cronbach alpha (α) is used to assess indicator and construct dependability, respectively. Furthermore, the average variance extracted (AVE) is employed for convergent validity, Fornell and Larcker criterion, and HTMT ratio for discriminant validity. The FD, CR, and AVE values must all be ≥0.70 (4), but the AVE value must be ≥0.50 (47, 48). According to the Fornell and Larcker criterion, the square root of all diagonal values should be greater than the square root of off-diagonal values. HTMT results, on the other hand, should be close to zero but >0.85 (49).

## Data Analysis

The demographic factors are an important indicator to judge the sample characteristics. There are four demographic indicators are discussed by the respondents. In the total sample, there were 46.98% male and 53.02% were women. They belong to different age classes, such as 20 and fewer years, 21–25, 26–30, 31–35, 36–40, 41–45, and 46–50. Where 19.42, 31.76, 20.47, 14.17, 5.51, and 8.66% belong to each class, respectively. Respondents belong to four different educational qualifications. Where, 35.17% have Bachelor and lower qualifications, 25.72% (Master), 33.86% (Doctorate or Ph.D.), and 5.25% (Diploma and others qualification) as depicted in Table 1.

**Table 1:** Demographics of the respondents.

**Demographics**	**Respondents**	**%**
**Gender**		
Male	179	46.98%
Female	202	53.02%
**Age**		
20 and fewer years	74	19.42%
21–25	121	31.76%
26–30	78	20.47%
31–35	54	14.17%
36–40	21	5.51%
41–45	33	8.66%
46–50		
**Education**		
Bachelor and lower	134	35.17%
Master	98	25.72%
Doctorate	129	33.86%
Diploma and others	20	5.25%
**Industry**		
Economist	98	25.72%
Financial Analysts	125	32.81%
Managers	87	22.83%
Teachers	71	18.64%
**Total sample**	381	

### Model Measurement

**Figure 2:**
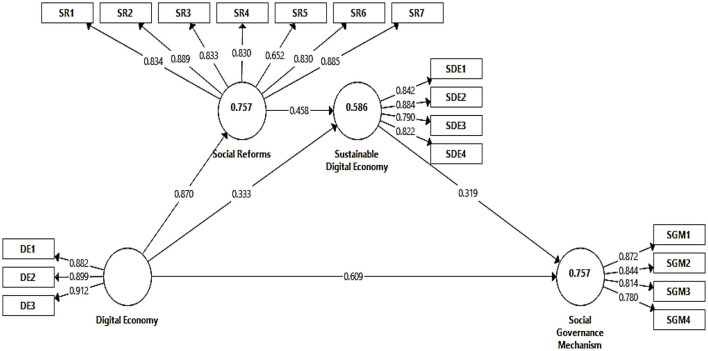
Model measurement.

The model measurement involves reliability and validity analysis. Table 2 depicts the overall measurement model. All the indicators used to estimate each variable are reliable as the factor loading for each construct is higher or equal to 0.70. Hence indicator reliability is maintained. The α and CR are considered to calculate the reliability of constructs in the model. The α-values are for the digital economy (0.880), sustainable digital economy (0.855), social governance mechanism (0.847), and social reforms (0.920). On the other hand, CR values are for the digital economy (0.926), sustainable digital economy (0.902), social governance mechanism (0.847), and social reforms (0.937). All these values for α and CR are above the threshold point 0.70 hence the construct reliability is satisfactory. Figure 2 illustrates the PLS-algorithm outputs.

**Table 2:** Model measurement and descriptive statistics.

**Constructs**	**Items**	**FD**	**α**	**CR**	**AVE**
Digital economy	DE1	0.882	0.880	0.926	0.806
	DE2	0.899			
	DE3	0.912			
Sustainable digital economy	SDE1	0.842	0.855	0.902	0.698
	SDE2	0.884			
	SDE3	0.790			
	SDE4	0.822			
Social governance mechanism	SGM1	0.872	0.847	0.897	0.686
	SGM2	0.844			
	SGM3	0.814			
	SGM4	0.780			
Social reforms	SR1	0.834	0.920	0.937	0.681
	SR2	0.889			
	SR3	0.833			
	SR4	0.830			
	SR5	0.652			
	SR6	0.830			
	SR7	0.885			

*DE, digital economy; SDE, sustainable digital economy; SGM, social governance mechanism; SR, social reforms*.

The AVE value estimates the convergent validity, hence the AVE values for all constructs above the 0.50 threshold. It indicated that the convergent validity is maintained for all variables in the model.

The discriminant validity is measured through Fornell and Larcker ratio and HTMT ratio. Where Table 3 represents the output of the Fornell Larcker criterion where the square root of all diagonal values are above the off-diagonal of correlation values hence the outcomes are satisfactory. Therefore, multicollinearity issues or discriminant validity issues were found, and all constructs are discriminately valid.

**Table 3:** Fornell and Larcker criterion.

	**Digital economy**	**Social governance mechanism**	**Social reforms**	**Sustainable digital economy**
Digital economy	0.898			
Social governance	0.843	0.828		
mechanism				
Social reforms	0.870	0.831	0.825	
Sustainable digital economy	0.731	0.764	0.748	0.835

The HTMT ratio output is presented in Table 4 and all values are near to zero and <0.85 threshold point for HTMT ratio. Hence, the second measure of discriminant validity also confirmed that no such discriminant validity issues were found.

**Table 4:** HTMT ratio.

	**Digital economy**	**Social governance mechanism**	**Social reforms**	**Sustainable digital economy**
**Digital economy**			
Social governance mechanism	0.571	–		
Social reforms	0.359	0.532	–	
Sustainable digital economy	0.636	0.814	0.441	–

### Structural Mode and Hypothesis Testings

The structural model assessment was carried out to test the hypothesis and check the relationships between constructs in the model. Overall, the study proposes five direct effects and three indirect effects or a total of eight hypotheses. Direct effects are illustrated in Table 5.

**Table 5:** Direct effects.

**H**	**Paths**	**Original sample**	**Sample mean**	**Standard deviation**	***T* statistics**	***P*-values**	***R* ^2^**	**Results**
H1	Digital economy → Social governance mechanism	0.609	0.609	0.038	15.971	0.000	0.757	Accepted
H2	Digital economy → Sustainable digital economy	0.333	0.333	0.056	5.937	0.000	0.586	Accepted
H3	Sustainable digital economy → Social governance mechanism	0.319	0.319	0.037	8.652	0.000		Accepted
H4	Digital economy -> Social reforms	0.870	0.870	0.015	57.168	0.000	0.757	Accepted
H5	Social reforms → Sustainable digital economy	0.458	0.459	0.058	7.936	0.000		Accepted

The digital economy has a positive and significant impact on social governance mechanisms. The output *t statistics* 15.971, *p* 0.000, confirmed that *H1* is accepted. The digital economy has a meaningful impact on the sustainable digital economy where *t statistics* 5.937, *p* 0.000 confirmed that *H2* is accepted. Likewise, a sustainable digital economy has a positive impact on social governance mechanism as output *t statistics* 8.652, *p* 0.000, hence the *H3* is accepted. The *H4* is also accepted as *t statistics* 57.168, *p* 0.000 confirmed that digital as the digital economy rises the social reforms also increases and both have a positive relationship. The last direct effect confirmed that social reforms have a positive impact on sustainable digital economy as *t statistics* 7.936, *p* 0.000. Hence, *H5* was accepted. *R*^2^ values demonstrated that 75.7, 58.6, and 75.7% predictive impact of independent variables on the dependent variables.

**Figure 3:**
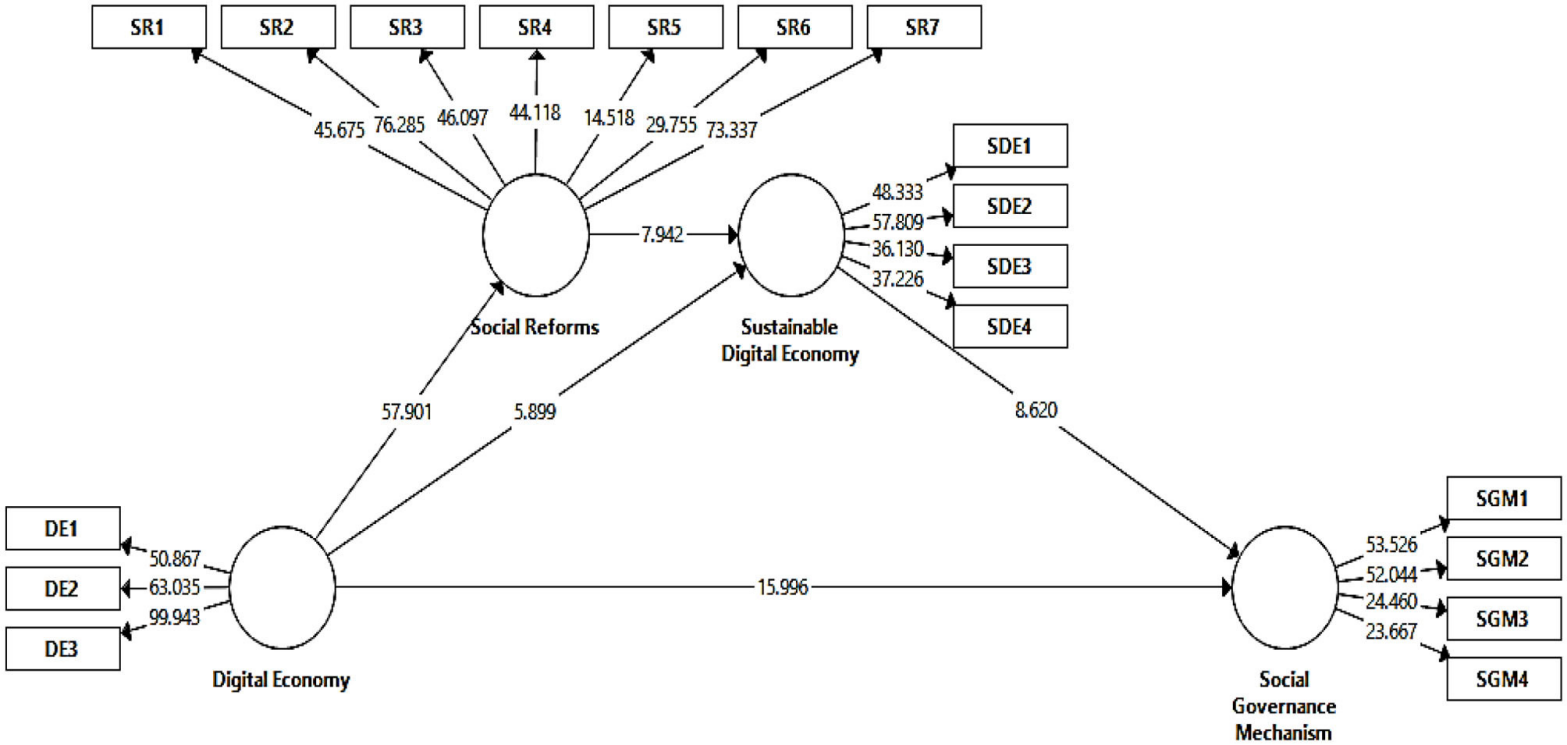
Structural model.

Indirect effects or mediating analyses are presented in Table 6. Social reforms confirmed partial mediation between the digital economy and sustainable digital economy as *t statistics* 7.817, *p* 0.000. Therefore, H6 accepted. The sustainable digital economy also proved a partial mediation between the digital economy and social governance mechanism where *t statistics* 5.853, *p* 0.000, hence *H7* accepted. Finally, the last indirect effect is also accepted as there exists a significant serial or chain mediation between digital economy, social reforms, sustainable digital economy, and social governance mechanism as *t statistics* 4.839, *p* 0.000. Figure 3 showed the output of t-statistics, and it is the outcome of PLS-bootstrapping.

**Table 6:** Indirect effects.

**H**	**Paths**	**Original sample**	**Sample mean**	**Standard deviation**	***T* statistics**	***P*-values**	**Results**
H6	Digital economy → Social reforms → Sustainable digital economy	0.399	0.399	0.051	7.817	0.000	Accepted
H7	Digital economy → Sustainable digital economy → Social governance mechanism	0.106	0.106	0.018	5.853	0.000	Accepted
H8	Digital economy → Social reforms → Sustainable digital economy → Social governance mechanism	0.127	0.128	0.026	4.839	0.000	Accepted

## Discussion

The digital economy plays a vital role in developing a strong social governance mechanism. This research explores the impact of the digital economy on social governance mechanisms. In addition, it investigates the mediating role of a sustainable digital economy and social reforms. This study found that the digital economy positively impacts social reforms, sustainable digital economy, and social governance mechanisms. It indicates that the digital economy's prosperity enhances the sustainable digital economy (50). Green innovation such as green bonds is sustainable and socially important innovations important for social governance (8). Likewise, reforms such as social and economic reforms are essential components of a sustainable digital economy (22, 45). The greater connectedness and networking that digital technologies provide, such as enhancing communication, services, and trade, is changing society (21). Policymakers are increasingly examining the original sustainability policy concepts in various national governments and international organizations such as the United Nations (UN) and the Organization for Economic Co-operation and Development (OECD) (21). Whilst growth of a digital economy may boost efficiency and benefit global and regional economies, it is also an indispensable tool for sustainability challenges in terms of social (i.e., the benefits or costs imposed by disruptive digital technologies on social networks and ways of life, including threats to economic sustainability and the rise of economic disparity) and environmental (i.e., natural resource stewardship and concern for future generations) well-being (21). In recent years, the digital economy and the green economy have become the most important topics on the environmental policy agenda. The first section of the article examines current thinking on the environmental impact of the digital economy, particularly on the special governance mechanism, while the second piece examines the green economy and social reforms. Both perspectives have risen to prominence in the fields of ITC policy and sustainable development (2, 3).

## Conclusion

The role of the digital economy in improving social governance mechanisms is indispensable. Moreover, sustaining a strong sustainable digital economy is a key component to shape up the social governance mechanism. It is important to investigate the role of the digital economy in shaping the impactful social governance system. The impact of the digital economy on social governance mechanisms is investigated in this research. It also includes indirect effects or mediating forces like social reforms and a long-term digital economy. The research is based on a positivist mindset and is a survey study. It's a cross-sectional investigation. Employees from four various professions, such as economists, financial analysts, managers, and teachers, served as the study's unit of analysis. The sampling method was random sampling, and the data was collected using a questionnaire. Structural equation modeling (SEM) was used as a data analysis technique. The findings of the research demonstrated that the digital economy has a good impact on the social governance mechanism. Similarly, the digital economy has a favorable impact on social changes and the long-term viability of the digital economy. Social reforms have also been shown to have a good relationship with a long-term digital economy. The indirect effects and structural model output revealed that social change partially mediated the digital economy and long-term digital economy sustainability. Furthermore, a sustainable digital economy validated a partial mediation between the digital economy and social governance mechanisms. Finally, the analysis revealed that the digital economy, social reforms, long-term digital economy, and social governance mechanism are all intertwined. To establish a strong social governance mechanism, policymakers and government agents should work on improving the digital economy.

The implication of study is that management can influence knowledge transfer powerfully by launching non-market, intrinsic incentives (51) that “allow for the establishment of psychological contracts related to emotional loyalties,” which in turn increases individuals' motivation to share knowledge (52). Individuals may feel a sense of appreciation or professional and personal progress as a result of the successful implementation of these social governance processes. These techniques establish an environment of identification, trust, and commitment that is devoid of the “perfunctory compliance” that comes with hierarchical control (53). Therefore, applying sustainable digital economy and digital economy through social reforms serves to strengthen the social governance mechanism.

## Limitation of the Study

There are few research limitations in current research. Firstly, this paper investigates sustainable digital economy and social reform as mediators in the current research model. Secondly, the current research model has the paucity of a moderating role that may buffer the current association among constructs. Thirdly, the paper is cross-sectional and the findings of the research are purely based on the response of respondents only. Therefore, it may produce a bias in the overall findings of the research. These research limitations open several notable research avenues. Future scholars should add moderator and mediating variables in the model. Additionally, the same model should be tested in other country settings to increase the generalizability of the findings. Future scholars should conduct longitudinal research on the topic to gain an depth understanding of the findings.

## Data Availability Statement

The original contributions presented in the study are included in the article/supplementary material, further inquiries can be directed to the corresponding author.

## Ethics Statement

All subjects gave their informed consent for inclusion before they participated in the study. The study was conducted in accordance with the Declaration of Helsinki and the protocol was approved by the Henan Police College, China.

## Author Contributions

FN: conceived, designed, wrote the paper, read, and agreed to the published version of the manuscript.

## Conflict of Interest

The author declares that the research was conducted in the absence of any commercial or financial relationships that could be construed as a potential conflict of interest.

## Publisher's Note

All claims expressed in this article are solely those of the authors and do not necessarily represent those of their affiliated organizations, or those of the publisher, the editors and the reviewers. Any product that may be evaluated in this article, or claim that may be made by its manufacturer, is not guaranteed or endorsed by the publisher.
